# Relationship between Serum Leptin, Ghrelin and Dietary Macronutrients in Women with Polycystic Ovary Syndrome 

**DOI:** 10.22074/ijfs.2015.4546

**Published:** 2015-10-31

**Authors:** Bahram Pourghassem Gargari, Shiva Houjeghani, Laya Farzadi, Sheyda Houjeghani, Abdolrasoul Safaeiyan

**Affiliations:** 1 Nutrition Research Center, Department of Biochemistry and Diet Therapy, Faculty of Nutrition, Tabriz University of Medical Sciences, Tabriz, Iran; 2 Students’ Research Committee, Faculty of Nutrition, Tabriz University of Medical Sciences, Tabriz, Iran; 3 Women’s Reproductive Health Research Center, Tabriz University of Medical Sciences, Tabriz, Iran; 4 Department of Obstetrics and Gynecology, Faculty of Medicine, Tabriz Azad University of Medical Sciences, Tabriz, Iran; 5 Department of Statistics and Epidemiology, Faculty of Health, Tabriz University of Medical Sciences, Tabriz, Iran

**Keywords:** Leptin, Gherkin, Habitual Diet, Polycystic Ovary Syndrome

## Abstract

**Background:**

Polycystic ovary syndrome (PCOS) is the most common endocrinopathy in women. It may involve an impairment in physiologic regulation of leptin and ghrelin. There is limited, controversial data on the relation of dietary components with leptin and ghrelin in PCOS, so the current study has been conducted to explore the effects of different macronutrients on serum levels of leptin and ghrelin in PCOS and healthy subjects.

**Materials and Methods:**

In this case-control study, we randomly choose 30 PCOS pa- tients and 30 healthy age and body mass index (BMI) matched controls. Intake of macronutrients [protein, total fat, saturated, monounsaturated and polyunsaturated fatty acids (PUFA), carbohydrate, dietary fiber] and energy were assessed using 3-day, 24-hour food recall and food frequency questionnaires (FFQ). Fasting hormonal status was measured for each participant.

**Results:**

PCOS women had higher levels of serum leptin, insulin, testosterone, and luteinizing hormone (LH), whereas sex hormone-binding globulin (SHBG) was lower compared to healthy women. There was no significant difference in mean ghrelin concentrations between the groups. Among PCOS women, independent of BMI and total energy intake, we observed an inverse association between leptin concentration and total dietary fat (β=-0.16, P<0.05) and saturated fatty acid (SFA) intake (β=-0.58, P<0.05). This relationship was not seen in the healthy subjects. There was no significant association between ghrelin and macronutrients in PCOS and healthy participants.

**Conclusion:**

Certain habitual dietary components such as fat and SFA may decrease serum leptin, whereas ghrelin is not influenced by these in PCOS women. More studies are needed to better clarify the effects of dietary macronutrients on serum leptin and ghrelin.

## Introduction

Polycystic ovary syndrome (PCOS) is a heterogeneous collection of signs and symptoms that form a spectrum of a mild disorder in some but a severe disturbance of reproductive endocrine and metabolic functions in others. PCOS is diagnosed by amenorrhea/oligomenorrhea, clinical or biochemical signs of hyperandrogenism and/ or polycystic ovaries. It is one of the most common endocrinopathies. The prevalence of PCOS has risen substantially, from 6-8% to 12-20%, as a result of the adoption of the Rotterdam criteria for diagnosis. This criteria has introduced the following different phenotypes: classic (characterized by hyperandrogenism and oligoanovulation, with or without PCO morphology, and corresponding to the previous National Institutes of Health definition), ovulatory (hyperandrogenism and PCO), and normoandrogenic (oligoanovulation and PCO) (1-4).

Leptin, a 167-aminoacid peptide hormone, is a product of the human obese (*OB*) gene. It is related to the circulatory system by adipose tissue as a function of energy stores (5). Leptin has a number of important effects on eating behavior, energy expenditure and body weight (6, 7). Studies have indicated leptin’s role in reproduction and its involvement in the regulation of gonadotropin- releasing hormone (GnRH), follicle stimulating hormone (FSH), luteinizing hormone (LH), adrenocorticotropic hormone (ACTH), cortisol, and growth hormone (GH) concentrations. Leptin plays a major role in interactions that take place between nutritional status of the body and the hypothalamic-pituitary-ovarian axis (8-10).While data in other groups is high, there is relatively little information about the influence of specific dietary factors on circulating leptin concentrations among PCOS women. A study has shown that reduced carbohydrate intake rather than reduced fat or protein intake lowered serum leptin in obese subjects (11). Total fat and polyunsaturated fatty acid (PUFA) intakes had a positive association with plasma leptin level in American men with normal weight (12). Reduction of 24-hour circulating leptin concentrations in women by the consumption of high-fat meals was reported (13). Intake of dietary fiber showed an inverse association with serum leptin in a group of young Japanese women (14). Leptin had a positive relation with saturated fatty acids (SFA) in healthy women (15). Another study reported a negative association of leptin with energy intake from carbohydrates and a positive association with energy from dietary fat in healthy subjects (16).

Ghrelin is an acylated 28 amino acid peptide originally isolated from the rat stomach and later found in the intestines, pancreas, testes, and ovaries. Ghrelin enhances the appetite, stimulates food intake, and reduces fat utilization. It is involved in ovarian function. Its low levels are probably associated with insulin resistance and positive energy balance or obesity (17-22). Studies have shown impaired homeostasis of ghrelin in PCOS patients (22, 23). This situation may disturb the normal relation of different dietary factors and serum ghrelin levels, resulting in an altered food intake pattern and energy homeostasis. Studies on the effect of various dietary factors on fasting ghrelin levels in PCOS patients are limited and have conflicting results. A recent study on PCOS women has shown that protein intake suppressed postprandial ghrelin significantly longer compared to glucose (24). Another study on obese and overweight postmenopausal women with elevated serum insulin levels showed a negative association between dietary fat and carbohydrates with leptin. This association was positive with ghrelin (25).

With attention to the scarcity of data and conflicting results of studies on PCOS women, the objectives of this study were to examine the relationship between habitual dietary components (protein, total fat, saturated, monounsaturated and PUFA, carbohydrates and dietary fiber) with these peptides in PCOS women compared to healthy women.

## Materials and Methods

### Study participants

This case-control study enrolled 30 PCOS patients and 30 healthy age and body mass index (BMI) matched controls at Alzahra Hospital, Tabriz, Iran, from 2009 to 2010. The study participants were selected from an outpatient setting in the Gynecology and Obstetrics Ward. Participants were selected by the accidental (convenience) sampling method.

After being informed on the purpose and procedures of the study, all subjects signed an informed consent. Diagnosis of PCOS was made by a gynecologist using Rotterdam criteria (4). The study protocol was approved by the Ethics Committee at Tabriz University of Medical Sciences. Health status of the control group women were determined by medical history, physical and pelvic examinations and complete blood test. Control group participants had no signs of PCOS such as hyperandrogenism, menstrual dysfunction or polycystic ovaries.

Exclusion criteria for all subjects included pregnancy, hypothyroidism, hyperprolactinemia, Cushing’s syndrome, congenital adrenal hyperplasia, current or previous (within the last 3 months) use of oral contraceptives, glucocorticoids, anti-androgens, ovulation induction agents, anti-diabetic and anti-obesity drugs or other hormonal drugs.

### Dietary intake and anthropometry

Dietary intake and habits were assessed using 3-day 24-hour food recall and food frequency (FFQ) questionnaires. Participants were asked to recall the type and amount of food and beverage consumed using standard household measures (cups, tablespoons, etc.). The interviewers reviewed the questionnaire entries with the subject in order to clarify servings, recipes and forgotten foods. Food intake data obtained from the PCOS and healthy control groups were analyzed for protein, total fat, SFA, monounsaturated fatty acids (MUFA), PUFA, carbohydrates, dietary fiber and energy using nutritionist Ш diet analysis software.

In each woman we measured weight and height to calculate BMI. Body height was measured to the nearest 0.1 cm with the subject standing without shoes. Body weight in light indoor clothing was measured to the nearest 0.1 kg. The BMI was calculated using the standard formula of weight (kg)/height (m^2^).

### Biochemical assays

After undergoing a history and physical examination, venous blood sampling was performed for the hormonal assays. Blood samples were taken in the morning at 09:00 hours after a 12-hour overnight fast. The serum was separated and frozen at -70˚C until assayed.

The analyses were carried out during the early follicular phase in women who had menstrual cycles and in any phase of the cycle in PCOS patients. Serum leptin levels were measured using the Human Leptin ELISA kit (BioVendor GmbH, Im Neuenheimer Feld 583, D-69120 Heidelberg, Germany). In all subjects we measured plasma immunoreactive ghrelin levels with a commercially available radioimmunoassay that uses ^125^I-labeled bioactive ghrelin as a tracer and a rabbit polyclonal antibody raised against full-length octanoylated human ghrelin (Phoenix Parmaceuticals Inc., Belmont, CA, USA) that recognizes both acylated and des-acylated ghrelin.

Levels of serum LH and FSH were determined by the direct immunoenzymatic method [DiaMetra Srl, Folingo (PG) Italy]. The measurement of serum sex hormone-binding globulin (SHBG) was performed using an enzyme-linked immunosorbent assay (ELISA) kit (IBL Immuno-Biological Laboratories, Flughafenstrasse52A, D-22335, Hamburg, Germany). Total testosterone levels were determined using a commercially available ELISA kit (Monobind Inc., Lake Forest, CA, USA).

### Statistical analyses

Results are expressed as mean ± SD. Comparisons between two groups were made using the independent samples t test. Pearson correlation analyses were performed to define correlations between parameters. Simple regression modeling was applied to analyze the impact of independent variables on dependent variables. Statistical evaluations were performed by running the SPSS/PC software package (SPSS, Inc., Chicago, IL, USA). P values of less than 0.05 were regarded as statistically significant.

### Results

### Anthropometric and biochemical data

Baseline characteristic of the study groups are presented in table 1. We observed no significant difference in BMI between the two study groups. Analysis of biochemical data showed significantly higher mean leptin concentrations in PCOS women compared to healthy subjects. There was no significant difference in mean ghrelin concentrations between the two groups.

Analysis of dietary variables revealed dietary energy intake was lower in the PCOS group (1334.9± 143.4 kcal/d) compared to controls (1716.1 ± 142.07 kcal/d, P=0.007), but the relative macronutrient contribution to the daily energy intake did not differ between groups (Table 1).

**Table 1:** Anthropometric and biochemical data of study participants


	PCOS (n=30)	Controls (n=30)

Age (Y)	25.83 ± 4.00	26.06 ± 4.44
Height (cm)	160.1 ± 6.01	162.4 ± 6.52
Weight (kg)	64.4 ± 10.46	62.4 ± 8.82
BMI (kg/m^2^)	25.00 ± 3.61	23.68 ± 3.07
Leptin (ng/ml)	21.68 ± 4.49**	17.96 ± 0.54
Ghrelin (pmol/l)	210.33 ± 58.50	216.00 ± 80.84
Insulin (mu/l)	14.91 ± 1.78^*^^*^	7.90 ± 1.16
Total testosterone (ng/ml)	0.75 ± 0.60^*^	0.45 ± 0.26
SHBG (ng/ml)	31.81 ± 14.29^*^	52.34 ± 23.41
LH (mIU/ml)	12.50 ± 2.33^*^^*^	4.86 ± 2.12
FSH (mIU/ml)	6.03 ± 1.64	5.74 ± 1.10
Nutrient intake		
Energy intake (kcal/d)	1334.9 ± 143.4*	1716.1 ± 142.07
Carbohydrate (g/d)	171.6 ± 9.3	222.57 ± 20.4
Fat (g/d)	50.72 ± 2.72*	65.73 ± 6.24
Protein (g/d)	49.9 ± 2.43*	67.28 ± 5.89
Total dietary fiber (g/d)	6.0 ± 1.0	6.7 ± 0.6
Energy from carbohydrates (%)	51.13 ± 8.86	51.76 ± 8.67
Energy from fat (%)	34.06 ± 8.41	32.86 ± 6.55
Energy from protein (%)	14.8 ± 3.01	15.36 ± 3.45


Data are presented as means ± SD. BMI; Body mass index, FSH; Follicle stimulating hormone, LH; Luteinizing hormone, PCOS; Polycystic ovary syndrome, SHBG; Sex hormone-binding globulin, *P<0.05 with unpaired t test and **P<0.001 with unpaired t test.

### Correlation of habitual dietary intake with serum leptin and ghrelin levels

We observed a positive, significant association between leptin and BMI in the PCOS and control groups (Table 2). The PCOS group had a significant negative correlation between total dietary fat, MUFA and SFA and leptin concentration. However no significant correlation was found between dietary intakes and leptin levels in the control group. There was no significant association observed for ghrelin with dietary variables in either group.

The findings from bivariate correlation analyses were further examined using linear regression analysis in order to control for potential confounding factors. The unadjusted model (model 1), energy adjusted model (model 2) and fully adjusted model (model 3) for the association between main macronutrients (carbohydrate, protein, fat) and leptin, and fully adjusted model for the association between macronutrient subtypes (total fiber, water soluble and insoluble fiber, PUFA, MUFA, SFA) and leptin are presented. In the PCOS group, there was a statistically inverse association between fat intake and leptin (β=-0.43, P=0.02) which remained significant after adjustments for BMI and total energy intake (β=-0.16, P=0.04). This suggested that for every one gram increase in fat intake, there was a 0.226 ng/ml decrease in leptin among PCOS women who were similar in energy intake and BMI. For other macronutrients and macronutrients subtypes, there was a significant inverse association between SFA intake and serum leptin levels after adjusting for BMI and energy intake (β=-0.579, P=0.036). In healthy subjects, we did not observe a significant association between nutrients and leptin concentration (Table 3).

Ghrelin relationships with dietary factors are shown in table 4. We used three models to assess the ghrelin relationship with dietary factors. Model 1 presents unadjusted, model 2 shows the adjusted model (controlling for energy intake), and model 3 shows the adjusted model (controlling for both energy intake and BMI). Overall, there were no statistically significant associations between ghrelin and macronutrients or macronutrients subtypes in the two study groups. The association between carbohydrate intake and serum ghrelin was near to significant (β=-0.7, P<0.1).

**Table 2:** Pearson correlation tests of dietary variables with leptin and ghrelin concentrations.


	PCOS (n=30)		Controls (n=30)	
	Leptin	Ghrelin	Leptin	Ghrelin

Age (Y)	0.05	0.49	0.11	0.05
Weight (kg)	0.74**	-0.24	0.80**	-0.24
BMI (kg/m^2^)	0.84**	-0.04	0.93**	-0.22
Total energy intake (kcal/d)	0.09	-0.17	0.10	-0.01
Carbohydrate (g/d)	0.18	-0.10	-0.03	0.08
Protein (g/d)	-0.002	-0.27	0.21	-0.04
Fat (g/d)	-0.43*	-0.05	0.18	-0.11
Total fiber (g/d)	-0.10	0.17	0.23	-0.16
Water soluble fiber (g/d)	0.17	-0.22	0.20	-0.24
Insoluble fiber (g/d)	0.13	-0.05	0.21	-0.08
PUFA (g/d)	-0.08	-0.13	0.12	-0.10
MUFA (g/d)	-0.37*	0.01	0.23	-0.16
SFA (g/d)	-0.54*	-0.40	0.15	-0.11


BMI; Body mass index, MUFA; Monounsaturated fatty acids, PCOS; Polycystic ovary syndrome, PUFA; Polyunsaturated fatty acids, SFA; Saturated fatty acids, *; P<0.05 with correlation test and **; P<0.001 with correlation test.

**Table 3:** Relationship between leptin concentration to macronutrient and macronutrient subtypes in study groups according to regression analysis


	PCOS (n=30)			Control (n=30)		
	Model	β	P value	Model	β	P value

CHO (g/d)	1^a^	0.014	0.947	1^a^	-0.135	0.522
	2^b^	-0.219	0.522	2^b^	0.312	0.573
	3^c^	0.158	0.195	3^c^	0.073	0.382
Fat (g/d)	1^a^	-0.431	0.02	1^a^	-0.18	0.362
	2^b^	-0.556	0.02	2^b^	0.360	0.270
	3^c^	-0.160	0.04	3^c^	-0.130	0.504
Protein (g/d)	1^a^	0.096	0.657	1^a^	0.268	0.501
	2^b^	0.084	0.699	2^b^	0.410	0.344
	3^c^	-0.014	0.907	3^c^	0.037	0.807
**Macronutrient subtypes**						
Total fiber (g/d)	3^c^	0.169	0.373	3^c^	0.235	0.211
Water-soluble fiber (g/d)	3^c^	0.177	0.355	3^c^	0.126	0.588
Insoluble fiber (g/d)	3^c^	0.133	0.484	3^c^	0.140	0.548
PUFA (g/d)	3^c^	0.143	0.555	3^c^	-0.149	0.647
MUFA (g/d)	3^c^	-0.042	0.902	3^c^	0.500	0.367
SFA (g/d)	3^c^	-0.579	0.036	3^c^	-0.165	0.807


^a^; Unadjusted model, ^b^; Energy-adjusted model, ^c^; Energy- and body mass index (BMI) adjusted model, MUFA; Monounsaturated fatty acids, PCOS; Polycystic ovary syndrome, PUFA; Polyunsaturated fatty acids and SFA; Saturated fatty acids.

**Table 4:** Relationship between ghrelin concentration to macronutrient and macronutrient subtypes in the study groups according to regression analysis


	PCOS (n=30)			Control (n=30)		
	Model	β	P value	Model	β	P value

Carbohydrate (g/d)	1^a^	0.040	0.881	1^a^	0.119	0.574
	2^b^	-0.589	0.085	2^b^	0.057	0.920
	3^c^	-0.701	0.095	3^c^	0.190	0.737
Fat (g/d)	1^a^	0.031	0.885	1^a^	-0.342	0.396
	2^b^	-0.259	0.266	2^b^	-0.401	0.534
	3^c^	-0.024	0.917	3^c^	-0.185	0.776
Protein (g/d)	1^a^	-0.297	0.205	1^a^	0.211	0.599
	2^b^	-0.329	0.127	2^b^	0.191	0.665
	3^c^	-0.276	0.246	3^c^	0.324	0.469
Macronutrient subtypes	
Total fiber (g/d)	3^c^	-0.101	0.596	3^c^	-0.159	0.400
Water-soluble fiber (g/d)	3^c^	-0.218	0.246	3^c^	-0.240	0.201
Insoluble fiber (g/d)	3^c^	-0.049	0.791	3^c^	-0.420	0.672
PUFA (g/d)	3^c^	-0.318	0.265	3^c^	0.074	0.813
MUFA (g/d)	3^c^	0.374	0.345	3^c^	-0.315	0.504
SFA (g/d)	3^c^	-0.217	0.479	3^c^	0.117	0.756


^a^; Unadjusted model, ^b^; Energy-adjusted model, ^c^; Energy- and body mass index (BMI) adjusted model, MUFA; Monounsaturated fatty acids, PCOS; Polycystic ovary syndrome, PUFA; Polyunsaturated fatty acids and SFA; Saturated fatty acids.

## Discussion

We took into consideration the metabolic and endocrine importance of leptin and ghrelin in PCOS patients and investigated the dietary predictors of these hormones in this study. The results indicated a significant, inverse association of habitual dietary fat and SFA with leptin concentrations in PCOS patients after adjustment for total energy intake and BMI. In healthy individuals we observed no significant association between dietary intakes and serum leptin concentration. Ghrelin was not influenced by habitual dietary components.

Studies on the association of dietary factors with leptin concentration were conducted on healthy, obese or other than PCOS subjects, with contradictory results.

Havel et al. (13) in a study on healthy and normal weight women showed that high fat meals reduced circulating leptin concentrations which supported the results of the current study. In another study by Larsson et al. (26) on 64 healthy postmenopausal women, the authors reported that leptin levels negatively correlated with total fat (r=-0.36, P=0.004) as well as SFA (r=-0.31, P=0.014).

Kong et al. (25) found that higher habitual intake of dietary fat was associated with lower leptin in overweight and obese postmenopausal women. They observed an inverse association between leptin concentration and percentage of energy from carbohydrates.

In contrast to these studies, other studies failed to show any association between dietary fat and its subtypes with serum leptin levels (11, 14, 27). Numerous studies showed a positive correlation between serum leptin and dietary fat intake (12, 15, 16). Others showed a different association between serum leptin and dietary fat in men and women (28, 29). However, these studies were all conducted healthy subjects or other than PCOS patients.

The difference among PCOS women and healthy controls in term of leptin and its association with habitual dietary fat and SFA might be explained by varying hormone levels such as testosterone and particularly insulin. In our study, there were higher testosterone and insulin levels observed in PCOS women compared to the control group.

Androgen excess plays an important role in the development of PCOS (30). A number of studies have shown the role of fat and fatty acids in androgen synthesis (31, 32). Insulin modifies the hormone - nutrient associations and has a potential impact in the regulation of both leptin and ghrelin (33, 34). Although inconsistent, a stimulating effect of insulin on OB gene expression has been reported in several studies (35, 36). Another factor is plasma free fatty acids. Plasma leptin is sensitive to free fatty acids. An *in vitro* study has shown that free fatty acids inhibit leptin transcription in adipocytes (37). Donahoo et al. (38) found that increasing free fatty acids in humans led to a decreased leptin concentration. The reduction of leptin secretion after high fat meals might be attributed to decreased insulin release, which resulted in lowered glucose metabolism in adipose tissue. The consumption of high fat meals which consequently decrease total leptin, could demonstrate the adipogenic effect of diets high in fats (13, 39).

The present study found no significant association between ghrelin concentration and habitual dietary macronutrients in PCOS and healthy controls. Similar results were obtained in a study on fasting and postprandial ghrelin levels in PCOS women (40). In contrast to our study, Kong et al. (25) studied overweight and obese postmenopausal women. They reported that habitual dietary intake of carbohydrate and fat were associated with higher ghrelin concentrations. Barber et al. (41) showed that ghrelin levels were suppressed by oral glucose in women with PCOS. Kasim-Karakas et al. (24) reported that in PCOS patients protein intake suppressed ghrelin longer than glucose.

In our study the negative association between carbohydrate intake and serum ghrelin was near to significant. Ghrelin fluctuations have been shown to inversely correlate with those of insulin, when insulin concentrations rise, ghrelin concentrations fall (42-44). It is generally accepted that carbohydrate is the most effective macronutrient for ghrelin suppression, because of its rapid absorption and insulin secreting effect. Protein induces a prolonged effect and fat exhibits weak ghrelin suppressing capacity (45).

Our study had some limitations. First, was the use of self-reported nutrient intake which has the potential for tremendous recall bias and underreporting. This was likely the case for the PCOS group that had a significantly lower total energy intake. In addition, single fasting hormone measures were more limited than a 24 hour profile or hormone response to given macronutrient test meals. The sample size of our study might be considered too low. Blood samples from PCOS women were collected at any time, hence it was not possible to determine which cycle stage they were at.

## Conclusion

The results of the current study showed that in PCOS patients some components of habitual dietary intake such as fat and SFA were inversely associated with serum leptin concentrations, independent of BMI and total energy intake. There was no significant association between habitual dietary intake of macronutrients or their subtypes and serum ghrelin concentrations.

Based on these findings, limiting food stuffs high in fat (especially SFA) would be beneficial in appropriate control and management of either the metabolic or endocrine status of PCOS patients.
